# Examining minimal important change of the Canadian Occupational Performance Measure for subacute rehabilitation hospital inpatients

**DOI:** 10.1186/s41687-021-00405-y

**Published:** 2021-12-20

**Authors:** Kanta Ohno, Kounosuke Tomori, Tatsunori Sawada, Ryuji Kobayashi

**Affiliations:** 1 Major of Occupational Therapy, Department of Rehabilitation, School of Health Science, Tokyo University of Technology, 5-23-22, Nishikamata, Ota-City, Tokyo 144-8535 Japan; 2 Department of Occupational Therapy, Graduate School of Human Health Sciences, Tokyo Metropolitan University, Tokyo, Japan

**Keywords:** Canadian Occupational Performance Measure, Minimal clinically important difference, Patient-reported Outcome, Subacute rehabilitation hospital, Response shift

## Abstract

**Background:**

The Canadian Occupational Performance Measure (COPM) is an individualized patient-reported outcome designed to evaluate the self-perceptions of a patient’s occupational performance. Our study aimed to examine the minimal important change (MIC) in inpatients undergoing subacute rehabilitation. The MIC values were calculated using the three different anchor-based analyses with the transition index as an external criterion; the mean change method (MIC_MeanChange_), the receiver operating characteristic (MIC_ROC_) analysis, and the predictive modeling method adjusted for the proportion of improved patients (MIC_adjust_). In this study, the MIC_adjust_ value was considered as the most valid statistical method. We recruited 100 inpatients with various health conditions from subacute rehabilitation hospitals. Data were collected twice: an initial assessment and a reassessment one month later. The systematic interview format (Five Ws and How) was used for both the initial and second assessments to prevent information bias (response shift).

**Results:**

Three patients who indicated deterioration on the transition index were excluded from all analyses, and 97 patients were analyzed in this study. The MIC_adjust_ values were 2.20 points (95% confidence interval 1.80–2.59) for the COPM performance score and 2.06 points (95% confidence interval 1.73–2.39) for the COPM satisfaction score. The MIC_MeanChange_ and MIC_ROC_ values were considered less reasonable to interpret because the proportions of the improved patients subgroup were more than 50% (82.5%).

**Conclusions:**

The MIC_adjust_ value estimates from this study can help detect whether the patients’ perceived occupational performance improved or did not change. The results support the multidisciplinary use of COPM in clinical practice and research on subacute rehabilitation inpatients.

## Background

Occupational therapy is a health profession that operates on the principles of client-centeredness [1]. The World Federation of Occupational Therapy states: “The outcomes are client-driven and diverse and measured in terms of participation, satisfaction derived from occupational participation and/or improvement in occupational performance” [2]. Occupational performance is a person’s ability to perform the required activities, tasks, and roles of daily living and is categorized into three occupational dimensions: self-care, productivity, and leisure [3]. Occupational therapists are bound to evaluate various aspects of their clients’ occupational performance, including the client’s own perceptions [4].

The Canadian Occupational Performance Measure (COPM) is a well-known patient-reported outcome measure (PROM) in rehabilitation [3], and is a patient-specific measure to identify and evaluate a patient’s occupation as something that the patient “wants to do, needs to do, or is expected to do” (i.e., occupational performance) [5, 6]. Through a semi-structured interview, patients prioritize up to five occupational problems that are the most urgent or important but difficult to perform [5, 6]. The patient then rates each of the identified problems by self-evaluating their current PERFORMANCE score (COPM-P) and SATISFACTION with the current performance score (COPM-S) [5, 6]. The COPM is flexible to use in various clinical settings without any target population-related limitations. It is used in over 40 countries and has been translated into more than 35 languages [5, 6]. The COPM has become a global gold standard for clinical research and rehabilitation practice.

Some researchers have reviewed the psychometric properties of the COPM in various situations, and reported good validity, reliability (test–retest), and responsiveness [7, 8]. In a prior systematic review [9], however, no measurement properties met the criteria of the COnsensus-based Standards for the selection of health Measurement INstruments (COSMIN) methodology [10, 11] because some studies indicated sufficient quality of evidence, while others were of poor quality. In particular, regarding responsiveness, the quality of evidence was inconsistent because only two of the ten included studies met the COSMIN criteria [12, 13]. In the COSMIN guidelines, responsiveness has been defined as “the ability of PROM to detect change over time in the construct to be measured” [14, 15]. Furthermore, interpretability is considered an important aspect in selecting PROMs, although it is not a measurement property [11]. Interpretability is defined as “the degree to which one can assign qualitative meaning (that is, clinical or commonly understood connotations) to a PROM’s quantitative scores or change in scores” [16]. The original manual of the COPM indicated that a change of 2.0 points or more is regarded as a clinically important change [5], however, the methodology for calculating points is unclear, and the target sample is not described in detail.

There are multiple methodological approaches to calculate the minimal important change (MIC) [17, 18]. Kjeken et al. examined the MIC using a distribution method in adult patients with ankylosing spondylitis [19]. Eyssen et al. [13] investigated the MIC in home-dwelling adults using the anchor-based method that compares the change score of PROM with some other measures of change, considered an anchor or external criterion [17]. There is often a range in the MIC estimates that varies across the target population and clinical study context, because the MIC depends on the characteristics of the target population and the context [20]. Although, the MIC should be calculated for each target population, no studies have investigated the MIC of COPM in subacute patients using scientific methods. Therefore, the main objective of our study was to document the variability of the MIC values of the COPM using a common anchor-based calculation for inpatients undergoing various diagnoses in subacute rehabilitation hospitals.

## Methods

### Study design and ethics

This multicenter prospective longitudinal study was conducted in subacute rehabilitation hospitals in Japan. The study was performed in accordance with the ethical approval of the Tokyo Metropolitan University Ethics Committee (20052). All participants provided written informed consent before participating in the study.

### Participants

Participants were selected from the occupational therapy departments of three subacute rehabilitation wards in Tokyo. The inclusion criteria for the patients were as follows: (a) admitted to subacute rehabilitation wards between July 2020 and March 2021 (9 months), (b) received client-centered occupational therapy from occupational therapists, (c) aged ≥ 20 years, (d) able to understand written or spoken Japanese, (e) deemed to not have a severe cognitive impairment from their scores on the mini-mental state examination ([MMSE], i.e., a score of 20 or higher), and (f) had not been diagnosed or suspected to have an intellectual disability or cognitive impairment, and were not medically/psychiatrically unstable (based on a review of participants’ medical history and medical chart).

The inclusion criteria of the occupational therapists were as follows: (a) had a minimum of at least six months of full-time clinical experience as an occupational therapist, (b) had attended educational sessions regarding scoring and interpretation of the COPM administered by the first author, (c) had read the COPM manual, and (d) had completed a total of 15 COPM pre-post administrations with patients.

### Canadian Occupational Performance Measure (COPM)

The COPM is an individual measure that captures a client’s self-perception of actual performance and satisfaction in everyday living [5, 21]. The clients were asked to rate COPM-P and COPM-S for each of the prioritized occupations using an ordinal 10-point scale where 1 = “not able to do it at all” and 10 = “able to do it extremely well” or where 1 = “not satisfied at all” and 10 = “extremely satisfied” [5]. We obtained the average performance score by summing the ratings for the performance score over the prioritized problems and dividing them by the number of occupations. The average satisfaction scores were calculated in a similar manner [5].

### Transition index

To derive the MIC values, we used a transition index [22, 23] as an anchor to capture the patient’s impression of change for each of the occupational performances identified by the COPM. The question was: “To what degree have you perceived a change in problems of each identified occupation since the initial assessment?” The transition index was graded on a 7-point ordinal scale, where “1 = totally diminished” and “7 = much worse.” The transition index describes the magnitude and direction of the change in perceived health status over a given period. Multiple studies have used the transition index as an external criterion for calculating the MIC [13, 17, 24]. With reference to a previous study by Eyssen et al., those answering “1 = totally diminished,” “2 = diminished,” or “3 = slightly diminished” for at least three of the five problems on the transition index were labeled “Improved” (e.g., responders), patients answering “4 = no change” were labeled “No change” (e.g., nonresponders). Similarly, patients who indicated deterioration (“5 = slightly worse,” “6 = worse,” or “7 = much worse”) for at least three of the five problems on the transition index were labeled “Deteriorated.” Because this study focused on determining cutoff points of improvement in self-perception of occupational performance prioritized on the COPM, patients who were labeled “Deteriorated” were excluded from all analyses [13]. The anchors’ validity was evaluated with Polyserial correlation coefficients between the transition index and the respective change scores of the COPM-P and COPM-S. The correlation between the change scores on the PROM and the anchor question should be at least 0.30 to assume validity of the anchor [25].

### Data collection

The patients were assessed twice by occupational therapists who were bound to evaluate various aspects of their clients’ occupational performance, including the client’s own perceptions of the same occupational therapist in the initial assessment (T1, within 1 week from admission to the hospital), and the reassessment (T2). The patient and the occupational therapist planned the reassessment together about one month after the initial assessment or if the therapy was coming to an end before one month. In the first assessment, we collected the demographic characteristics of the participants, including information on health conditions (i.e., age, diagnosis, and sex) and MMSE. Further, years of experience of the occupational therapists were obtained from a staff database. COPM-P and COPM-S were assessed in both the initial assessment and reassessment. After the reassessment of the COPM was complete, patients were asked about the transition index.

### Measurement method of COPM to prevent response shift

To correctly interpret the longitudinal change score in PROMs, it must be assumed that the respondents’ perceptions remain stable over time; however, several studies have indicated that the meaning of patients’ self-evaluations may not be the same over time, a phenomenon called response shift [26–28]. Response shift is defined as “the change in the meaning of a person’s self-evaluation of a target construct (e.g., QOL) over time” [29]. There are three types of response shifts: a change in the meaning of one’s evaluation of a construct as a result of a change in one’s internal standards of measurement (recalibration), a change in one’s values (reprioritization), or a change in one’s definition of the construct (reconceptualization) [29]. If a response shift occurs in the measurement of the COPM, the MIC cannot be detected correctly.

The structural equation model (SEM) is an accurate statistical analysis for detecting the response shift effects of PROMs [28]. However, the COPM is a PROM classified as "Individualized", and allows the participants to select their own personal concerns, unlike the measurements that use predetermined personal concerns in the responder's list of questionnaire items [30]. Hence, we thought that it was difficult to apply SEM to investigate the response shift of the COPM, because the items of the COPM are determined by the respondents. In this study, we modified the COPM interview to decrease the impact of the response shift effect, especially “recalibration.” We used a systematic format that identifies problems more specifically, the “Five Ws and How” questions with reference to the method of identifying patient-centered goal setting by Randall et al. [31]. These questions were: “Who does the occupation? (subject),” “When do you do the occupation? (duration/frequency),” “Where do you do the occupation? (place),” “Why do you do the occupation? (reason/purpose),” and “How do you do the occupation? (method/procedure/means).” This format was used to facilitate patients’ recall of their own internal standards of self-perceptions of identified occupations. At the reassessment, the therapists showed the patients their occupations identified at the first assessment using the “Five Ws and How,” before the patients scored the COPM.

### Statistical analysis

The normality of the distribution of continuous variables was tested using the Shapiro–Wilk test. We used the Mann–Whitney *U* test for the comparison of change scores between the initial assessment (T1) and the reassessment (T2), and for the between-group comparisons.

In this study, we used three different anchor-based methods to examine MIC: the mean change method (MIC_MeanChange_) [32], the receiver operating characteristic (ROC)-based method (MIC_ROC_) [16], and the anchor-based MIC based on predictive modeling method (MIC_predict_) [33].

In the mean change method, MIC_MeanChange_ is defined as the change score on the measure of interest in the subgroup of patients that reported to be “a little better” (minimal important improvement) on the anchor question [32]. In the ROC method, sensitivity, specificity, and Youden index were calculated for COPM-P and COPM-S, whereby the Youden index = sensitivity + specificity − 1 [34]. In the current study, the highest Youden index was considered to represent the optimal MIC_ROC_ value, which reflects the COPM change score that provides the optimal distinction between “Improved” and “No change.” The area under the curve (AUC) for the ROC represents the probability that a client will be correctly identified by the COPM as “Improved.” The AUC values can range from 0.5, which indicates that prediction equals that of pure chance, to 1.0, which implies perfect accuracy in distinguishing “Improved” from “No change” [35]. In our study, AUC values ≥ 0.90 was considered excellent accuracy, between 0.80 and 0.89 was considered good, between 0.70 and 0.79 was considered fair, and less than 0.70 was considered poor accuracy [36]. At this cutoff point (i.e., MIC_ROC_), the diagnostic accuracy parameters of the COPM, sensitivity, specificity, positive predictive value (PPV), negative predictive value (NPV), accuracy, positive likelihood ratio (PLR), and negative likelihood ratio (NLR) were calculated, and their range was estimated at a 95% confidence interval.

The predictive modeling approach is related to the predicted probability that a patient belongs to the “Improved” group based on the anchor [33]. The MIC_predict_ was determined by logistic regression analysis, with the observed change score of the COPM as independent variable and the TI anchor as dependent variable [33]. The MIC_predict_ is defined as the change score associated with a likelihood ratio of 1[33]. Recently, this approach has been considered more precise as compared to the ROC method (MIC_ROC_) [33].

When the proportion of the “Improved” patients differs from 50%, the MIC will be biased. If more than 50% of the patients show “Improved” occupational performance, the MIC will tend to overestimate. If the percentage of “Improved” was not equal to 50%, we applied a formula for the adjustment of proportions “Improved” to obtain MIC_adjust_ as a more accurate estimate of the MIC [33]. Therefore, in this study, we considered the MIC_adjust_ the most statistically accurate.

All statistical analyses were performed using EZR (Saitama Medical Center, Jichi Medical University, Saitama, Japan), which is a graphical user interface for R (The R Foundation for Statistical Computing, Vienna, Austria). More precisely, it is a modified version of R commander designed to add statistical functions frequently used in biostatistics [37].

## Results

### Participant population and identified occupations

A total of 100 patients were enrolled in this study during the nine-month recruitment period. On the transition index, 80 of the 100 clients were labeled “Improved” (80%), 17 were labeled “No change” (17%), and 3 (3%) were labeled “Deteriorated.” Clients labeled “Deteriorated” (n = 3) were excluded from all analyses, and finally, 97 patients were analyzed in the current study.

Demographic characteristics of the participants are presented in Table 1. Thirty-six patients were male (37.1%), and the mean age was 73.6 ± 12.6 years. The diagnostic categories were stroke (38.1%), orthopedic diseases (including hip fractures and spinal cord injury) (57.7%), and disuse syndrome (a condition that is caused due to lack of physical activity, secondary to pneumonia and coronary occlusion) (4.1%). Among the 97 patients, 400 occupational performance problems were identified through COPM interviews. The distributions of the three dimensions are shown in Table 1. The most frequently prioritized problems were related to self-care activities (n = 207, 51.8%). This was followed by the domains of productivity (n = 141, 35.3%) and leisure (n = 52, 13.0%). The most dominant occupational category was household arrangement (n = 133, 33.3%).

**Table 1:** Participants’ characteristics and identified occupations in the Canadian Occupational Performance Measure

Variable	Category	Mean or count		SD	MAX	MIN
*Patients (n = 97)*						
Males		36 (37.1%)				
Age		73.6	±	12.6	97	36
Diagnosis	Stroke	37 (38.1%)				
	Orthopedic disease	56 (57.7%)				
	Disuse syndrome	4 (4.1%)				
MMSE		27.2	±	2.6	30	20
FIM	Total	81.9	±	16.6	123	42
	Motor	54.1	±	14.0	90	23
	Cognitive	28.1	±	5.3	35	9

Domain	Aspect	Count				
*Occupations identified by the COPM (n = 400)*						
Self-care (n = 207, 51.8%)	Personal care	128 (32.0%)				
	Functional mobility	63 (15.8%)				
	Community management	16 (4.0%)				
Productivity (n = 141, 35.3%)	Paid/unpaid work	8 (2.0%)				
	Household arrangement	133 (33.3%)				
	Play/school	0 (0%)				
Leisure (n = 52, 13.0%)	Quiet recreation	16 (4.0%)				
	Active recreation	27 (6.8%)				
	Socialization	9 (2.3%)				
*Occupational therapist (n = 30)*						
Experience years		5.9	±	3.4	16	2

*MMSE* mini-mental state examination, *FIM* functional independence measure

### Scores of the COPM in comparison with anchor

The distribution and mean change scores of the COPM per TI category are provided in Table 2. Of the 400 occupations, TI3 (A little improved) was the most frequent (n = 145, 36.3%). The total percentage of TI1 to TI3 represented the "Improved" group was 76.8% (n = 307), which was higher than that of the "No change (TI4)" group (n = 88, 22.0%) and the "Deteriorated (TI5 to TI7)" group (n = 5, 1.3%).

**Table 2:** The distribution and mean change scores of the Canadian Occupational Performance Measure sorted by patients’ responses in the transition index

Occupations identified by the COPM (n = 400)				COPM-P change score			COPM-S change score		
Transition index		Number of occupation (%)		Mean		SD	Mean		SD
1	Much improved	45 (11.3)	307 (76.8)	4.27	±	2.84	4.62	±	2.89
2	Improved	117 (29.3)		3.94	±	2.82	3.86	±	3.00
3	A little improved	145 (36.3)		2.62	±	2.33	2.78	±	2.57
4	No change	88 (22.0)	88 (22.0)	1.09	±	2.72	0.89	±	2.56
5	A little worse	4 (1.0)	5 (1.3)	− 2.25	±	1.50	− 0.50	±	4.73
6	Worse	1 (0.3)		− 5.00	±	–	0.00	±	–
7	Much worse	0 (0.0)		–	±	–	–	±	–

*COPM-P* Canadian Occupational Performance Measure Performance score, *COPM-S* Canadian Occupational Performance Measure Satisfaction score

Table 3 shows the COPM score distribution for the “Improved” and “No change” groups. Comparing the initial assessment (T1) versus the reassessment (T2), COPM-P and COPM-S increased significantly only in the “Improved” group (p < 0.001). The change scores (T2-T1) for the COPM-P and COPM-S were significantly higher in the “Improved” group compared to the “No change” group (p = 0.005, p < 0.001, respectively).

**Table 3:** Results of the Canadian Occupational Performance Measure (n = 97)

Variable	Group	T1: Initial assessment				T2: Reassessment				T2-T1: Change score				T1 vs T2 *P*-value	T2-T1: Change score Improved vs No change *P*-value
		Mean		SD	95%CI	Mean		SD	95%CI	Mean		SD	95%CI		
COPM-P	Improved (n = 80)	4.1	±	2.3	(3.6–4.6)	7.1	±	1.9	(6.7–7.5)	3.2	±	2.1	(2.7–3.7)	< 0.001*	0.005*
	No change (n = 17)	3.0	±	2.1	(1.9–4.0)	4.4	±	2.5	(3.1–5.7)	1.4	±	2.1	(0.3–2.5)	0.068	
COPM-S	Improved (n = 80)	3.8	±	2.2	(3.3–4.3)	7.0	±	1.9	(6.6–7.4)	3.0	±	2.1	(2.5–3.5)	< 0.001*	< 0.001*
	No change (n = 17)	2.8	±	1.9	(1.8–3.8)	3.7	±	2.2	(2.6–4.9)	0.9	±	1.3	(0.2–1.5)	0.219	

*COPM-P* Canadian Occupational Performance Measure Performance score, *COPM-S* Canadian Occupational Performance Measure Satisfaction score, *95% CI* 95% confidence interval

*p < 0.01

**Fig. 1:**
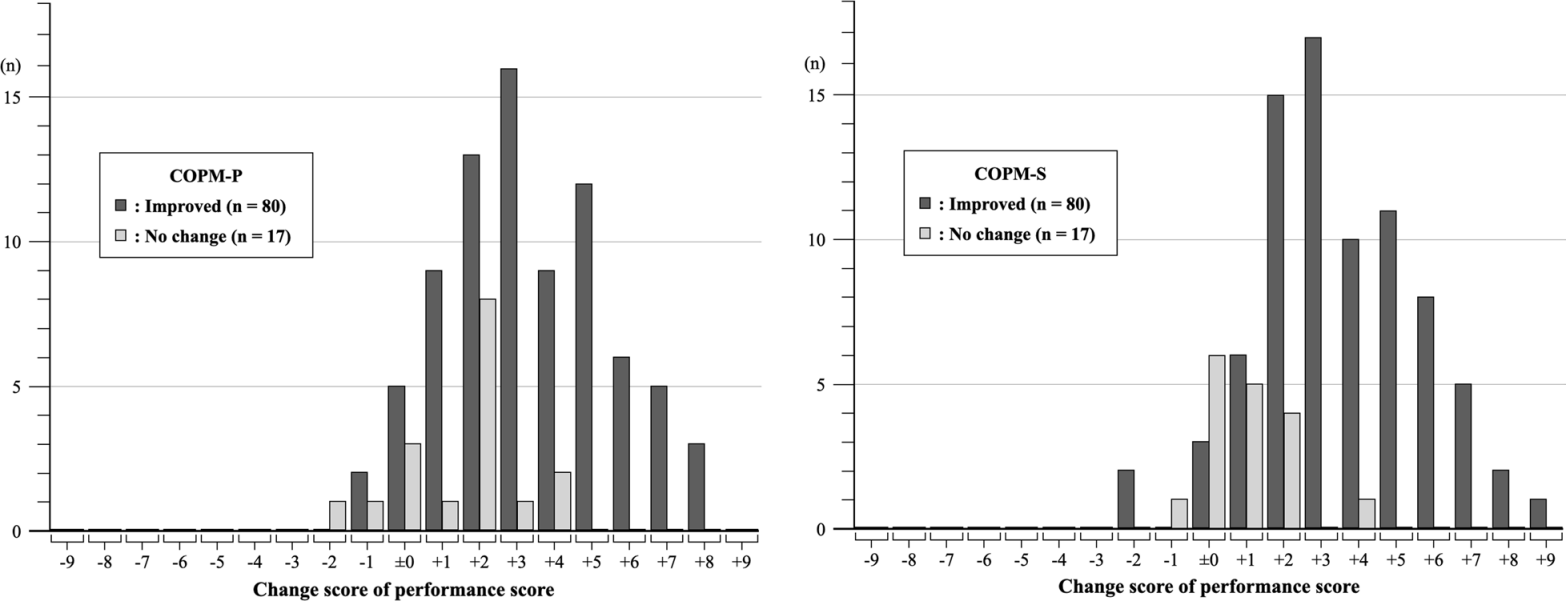
Histogram of the change scores of the Canadian Occupational Performance Measure Performance score and Satisfaction score

The frequencies of the change scores of COPM-P and COPM-S are presented in Fig. 1. Regarding the COPM-P, 3-point improvement (+ 3 points) was the most frequent (n = 16, 20.0%, MAX: + 7.6 points, MIN: − 1.8 points) in the “Improved” group, whereas 2-point improvement (+ 2 points) was the most frequent (n = 8, 47.1%, MAX: + 3.4 points, MIN: − 2.2 points) in the “No change” group. As for the COPM-S, 3-point improvement (+ 3 points) was the most frequent (n = 17, 21.3%, MAX: + 8.6 points, MIN: − 2.0 points) in the “Improved” group, whereas no change (± 0 point) was the most frequent (n = 6, 35.3%, MAX: + 3.6 points, MIN: − 1.8 points) in the “No change” group.

The correlations between anchor instrument and COPM change scores were 0.42 for the COPM-P and 0.66 for the COPM-S.

### MIC calculation: the mean change method (MIC_MeanChange_)

As shown in Table 4, the MIC_MeanChange_ values that represented the mean change score in participants who reported “A little better” on the transition index were 2.62 points and 2.78 points for COPM-P and COPM-S, respectively.

**Table 4:** The MIC values and diagnostic accuracy parameters of the COPM-P and COPM-S

Parameters	COPM-P			COPM-S		
	Estimate	95% confidence interval		Estimate	95% confidence interval	
		Lower	Upper		Lower	Upper
MIC_MeanChange_	2.62	2.24	3.00	2.78	2.36	3.20
MIC_ROC_	1.75	–	–	2.25	–	–
Area under the Curve	0.72	0.58	0.85	0.84	0.75	0.93
Sensitivity	0.70	0.59	0.80	0.66	0.55	0.76
Specificity	0.71	0.44	0.90	0.94	0.71	1.00
Positive Predictive Value	0.92	0.82	0.97	0.98	0.90	1.00
Negative Predictive Value	0.33	0.19	0.51	0.37	0.23	0.53
Accuracy	0.70	0.60	0.79	0.71	0.61	0.80
Positive Likelihood Ratio	2.38	1.12	5.04	11.26	1.67	75.90
Negative Likelihood Ratio	0.43	0.27	0.67	0.36	0.26	0.50
MIC_predict_	2.71	2.27	3.16	2.79	2.35	3.22
MIC_adjust_*	2.20	1.80	2.59	2.06	1.73	2.39

*COPM-P* Canadian Occupational Performance Measure Performance score, *COPM-S* Canadian Occupational Performance Measure Satisfaction score

*MIC_adjust_: adjusted MIC_predict_ for the proportion of improved patients

### MIC calculation: the ROC method (MICROC)

Table 4 presents the MIC of the COPM-P and COPM-S to detect whether the occupational performance “Improved” or “No change” was observed, and to compare the diagnostic accuracy parameters. The Youden indexes were highest when the COPM-P score (i.e., MIC_ROC_) was 1.75 points based on sensitivity of 0.70, specificity of 0.71, and COPM-S was calculated to be 2.25 points based on sensitivity of 0.66, specificity of 0.94 (Table 4). The MIC_ROC_ of the COPM-P was below the cutoff point (2.0 points) in the COPM manual [5]. In contrast, the MIC_ROC_ of COPM-S was above the suggested cutoff point in the COPM manual [5].

### MIC calculation: the predictive modeling method (MIC_predict_/MIC_adjust_)

We found that the MIC_predict_ values were 2.71 (95% CI 2.27, 3.16) points and 2.79 (95% CI 2.35, 3.22) points for COPM-P and COPM-S, respectively. The MIC_predict_ values were adjusted because the proportion of “Improved” patients was 82.5%, which was not equal to 50%. After adjusting for the proportion “Improved,” the MIC_adjust_ values were slightly decreased to 2.20 (95% CI 1.80, 2.59) points and 2.06 (95% CI 1.73, 2.39) points for COPM-P and COPM-S, respectively.

## Discussion

### Comparison with previous studies

This is the first study to propose estimates for the interpretation of MIC in COPM scores using three anchor-based methodologies. The MIC values varied depending on the MIC analysis methods. Eyssen et al. indicated that the optimal cutoff points (MIC_ROC_) of the COPM-P and COPM-S were 0.90 points (AUC: 0.85) and 1.45 points (AUC: 0.85), respectively, using the ROC curve analysis with transition index as an external standard [13]. Moreover, Tuntland et al. determined the MIC_MeanChange_ of COPM using a 5-point rating scale as an external standard [38], and Kjeken et al. calculated the measurement error (smallest detectable difference) of the COPM using the distribution method [19].

In our study, the MIC_MeanChange_ values were higher than MIC_ROC_ and MIC_predict_ values for both COPM-P and COPM-S (Table 4). However, the MIC_MeanChange_ estimates do not reflect a true threshold for minimal improvement because it is defined as the mean change score of the subgroup who reported being “a little better” [33]. The MIC_ROC_ values of the COPM-P and COPM-S were 1.75 and 2.25 points, respectively. As a result of ROC analysis, the cutoff value of COPM-P did not indicate sufficient predictive accuracy, with an AUC of 0.72 (95% CI 0.58–0.85) [17, 39], based on PPV of 0.92 and PLR of 2.38. On the other hand, the predictive accuracy of the COPM-S was good, with an AUC of 0.84 (95% CI 0.75–0.93) [17, 39], based on PPV of 0.98 and PLR of 11.26. In the COSMIN checklist, ROC analysis is recommended to assess the responsiveness for continuous scores, such as the COPM [10, 11].

However, the MIC will be biased and overestimated if more than 50% of the participants are perceived as “Improved” [33]. The MIC_predict_ can be adjusted when the proportions of improved patients differ from 50% (MIC_adjust_) [33]. Hence, the methodology of our research is based on statistical and academic criteria, and the MIC_adjust_ values were considered more suitable than MIC_MeanChange_ and MIC_ROC_ values.

Furthermore, target populations in all of the above studies were in the chronic phase or under stable conditions, including outpatients with various conditions [13], home-dwelling older adults [38], and home-dwelling patients with ankylosing spondylitis [19]. In contrast, inpatients with varying diagnoses in subacute rehabilitation hospitals were recruited for this study. Within two months after the onset of disabling diseases, patients in Japan are eligible for admission to a subacute rehabilitation hospital to receive early and intensive rehabilitation [40]. Therefore, it is suggested that the patients in this study can recover naturally due to their admission to the subacute hospital shortly after the onset of disabling diseases. To the best of our knowledge, this is the first study to calculate the MIC values of the COPM in subacute settings.

### Measurement accuracy of the COPM

The COPM is classified as “Individualized” in the types of PROM, which allows the participants to select their own personal concerns, unlike the measurements that use predetermined personal concerns in the responder’s list of questionnaire items [30]. In fact, Eyssen et al. reported that the concordance rate of prioritized problems using COPM between the first and second assessment with a time interval of seven days (SD 1.6, range 4–14) was only 66% [41]. Verkerk et al. also reported a concordance rate of 74%, similar to that reported by Eyssen et al. [42]. Because a patient’s perception may change over time, “recalibration,” a type of the response shift effect defined as “changes in the internal standards” [42] in the COPM, was likely to occur. Furthermore, regarding the scoring system of the COPM, some studies indicated that patients were not familiar with scoring on scales and had difficulty converting the self-evaluation of their occupational performance into a number [43–45]. In particular, difficulties in quantifying self-perception increase with age [46, 47].

The SEM is an accurate statistical analysis for detecting the response shift effects of PROMs [28]. However, it is difficult to adopt SEM to investigate the COPM because its items are determined depending on the respondents (i.e., classified as "Individualized") [30]. The *then* test is the formerly used method to detect the response shift effect, especially recalibration [48]. In the *then* test, patients are asked to retrospectively rate (*then* measure) the initial assessment at the time of reassessment. Since the reassessment and *then* measure are administered at the same time, these two types of ratings are considered to have been evaluated based on the same internal criteria [49]. However, patients were required to accurately remember how they were functioning in the past, because in the *then* test there is a possibility of the patient’s responses being affected by recall bias [48, 50]. In the current study, we applied a systematic interview format (i.e., “Five Ws and How”) to identify patients’ occupations in more detail. During the reassessment, patients were asked to rate their self-perception of occupational problems while referring to the identified occupations in the initial assessment, in order to minimize response shift effects and recall bias [51–53]. We believe that this format may help therapists and patients share occupational problems in greater detail and improve measurement accuracy. When researchers and clinicians apply the MIC results in this study, it is necessary to specify occupations using the same measurement methodology.

### Study limitation

There are four major limitations in this study that could be addressed in future research. First, in general, the MIC values probably vary among the characteristics of the target population (i.e., diagnoses, ages, and stage of disease); therefore, further studies need to be performed according to specific patient groups. The second limitation is small sample size. The smallish sample size may cause increasing standard deviations, therefore patient heterogeneity might negatively affect the measure’s ability to discriminate between “No change” and “A little improved.” The third limitation concerns the measurement error of the TI (anchor). In this study, the proportion of the patients who were shown as "Improved" on the transition index was not equal to 50%. It might be possible that patients did not accurately recognize a difference between "No change" and "A little improved" at TI. Finally, in this study, we used a systematic interview format, the “Five Ws and How” to reduce the response shift effects of the COPM. However, this interview technique was our original, and there is no research to examine the response shift when applying the “Five Ws and How.” Thus, further research is needed to show evidence reducing recall bias using this method.

## Conclusion

Our study aimed to examine MIC values to distinguish between inpatients in the subacute stage who have a minimal important change in COPM-P and COPM-S and those who have none, using three different methodologies. The MIC_adjust_ values were established to be 2.20 and 2.06 points for COPM-P and COPM-S, respectively. We used a systematic interview guide, the “Five Ws and How,” to identify more detailed and minimized response shift effects. These findings support the interpretation of the meaning of intervention outcomes and facilitate the goal-setting process.

## Data Availability

All data generated or analyzed during this study are included in this published article.

## Abbreviations

PROMs: Patient-reported outcome measures
COSMIN: COnsensus-based Standards for the selection of health Measurement INstruments
COPM: Canadian Occupational Performance Measure
COPM-P: Canadian Occupational Performance Measure Performance score
COPM-S: Canadian Occupational Performance Measure Satisfaction score
MIC: Minimal important change
MMSE: Mini-mental state examination
ROC: Receiver operating characteristic
AUC: Area under the curve
PPV: Positive predictive value
NPV: Negative predictive value
PLR: Positive likelihood ratio
NLR: Negative likelihood ratio
FIM: Functional independence measure
SD: Standard deviation
CI: Confidence interval
TP: True positive
FN: False negative
FP: False positive
TN: True negative
